# The relation between thrombus burden and early mortality risk in inpatients diagnosed with COVID-19-related acute pulmonary embolism: a retrospective cohort study

**DOI:** 10.1186/s12890-023-02647-6

**Published:** 2023-09-13

**Authors:** Umran Ozden Sertcelik, Erdem Ozkan, Ahmet Sertcelik, Aysegul Karalezli

**Affiliations:** 1 Ankara Bilkent City Hospital, Department of Chest Diseases, Ankara, Türkiye; 2 Ankara Bilkent City Hospital, Department of Radiology, Ankara, Türkiye; 3 Faculty of Medicine, Department of Public Health, Division of Epidemiology, Hacettepe University, Ankara, Türkiye; 4 Faculty of Medicine, Department of Chest Diseases, Ankara Yildirim Beyazit University, Ankara, Türkiye

**Keywords:** Pulmonary embolism, Clinical decision making, Mortality, Mastora score, 2019 Novel Coronavirus Disease

## Abstract

**Background:**

COVID-19-related acute pulmonary thromboembolism (APE) is associated with poor outcomes in patients with COVID-19. There are studies investigating the association between thrombus burden and high risk of early mortality in the pre-COVID-19 period. This study aimed to evaluate the relationship between clot burden and early mortality risk in COVID-19-related APE patients.

**Methods:**

In this single-center retrospective cohort study, the data of hospitalized adult patients followed up for COVID-19-related APE between April 1, 2020, and April 1, 2021, were electronically collected. A radiologist evaluated the computed tomography (CT) findings and calculated the Mastora scores to determine clot burden. The early mortality risk group of each patient was determined using 2019 the European Society of Cardiology guidelines.

**Results:**

Of the 87 patients included in the study, 58 (66.7%) were male, and the mean age was 62.5±16.2 years. There were 53 (60.9%) patients with a low risk of mortality, 18 (20.7%) with an intermediate-low risk, and 16(18.4%) with an intermediate-high/high risk. The median total simplified Mastora scores were 11.0, 18.5, and 31.5 in the low, the intermediate-low, and the intermediate-high/high-risk groups, respectively (*p = 0.002*). With the 80.61% of *post-hoc* power of the study, intermediate-high/high early mortality risk was associated statistically significantly with the total simplified Mastora score (adj OR = 1.06, 95%CI = 1.02–1.11,p *= 0.009*). Total simplified Mastora score was found to predict intermediate-high/high early mortality risk with a probability of 0.740 (95% CI = 0.603–0.877): At the optimal cut-off value of 18.5, it had 75.0% sensitivity, 66.2% specificity, 33.3% positive predictive value, and 92.2% negative predictive value.

**Conclusions:**

The total simplified Mastora score was found to be positively associated with early mortality risk and could be useful as decision support for the risk assessment in hospitalized COVID-19 patients. Evaluation of thrombus burden on CT angiography performed for diagnostic purposes can accelerate the decision of close monitoring and thrombolytic treatment of patients with moderate/high risk of early mortality.

## Background

Acute pulmonary thromboembolism (APE) is a clinical phenomenon resulted from seen thrombi, usually formed in the deep veins of the lower extremity, reaching the lungs through venous blood flow. APE is a life-threatening serious condition with a fatality risk varying between 14% and 36%, depending on the severity of the disease [1, 2]. Early diagnosis and identification, and timely initiation of appropriate treatment can reduce early mortality risk by 2-to-10% [3]. Therefore, early estimation of the APE severity and risk classification is vital to determine the best treatment strategy. International guidelines [4] include several laboratory- and imaging-based clinical prognostic markers and scores, such as risk stratification of acute pulmonary embolism, pulmonary embolism severity index (PESI) score, and clinical parameters, such as hypotension and the ratio of the right ventricular diameter to the left ventricular diameter.

Computed tomography (CT) pulmonary angiography (CTPA) is widely used to diagnose APE, with localization of thromboembolism in the pulmonary vascular tree and estimation of the thrombus burden [5]. Quantitative pulmonary clot burden scoring systems (like simplified Mastora score) have been developed based on the localization of thromboembolism in the pulmonary vascular bed and the degree of occlusion [6–8]. However, evidence on association of these scores with clinical parameters indicating the severity of the disease and mortality is scarce and inconclusive [9, 10].

COVID-19 can lead to prothrombotic state through activation of the coagulation system and endothelial dysfunction over time [11]. Thus venous thromboembolism and arterial thrombosis are among the most serious complications of this disease [12]. The most common thrombotic manifestation of COVID-19 is APE, which increases the length of hospital stay, morbidity, and mortality [13].Yet, data on prognosis of the thrombus burden and localization of embolism in COVID-19-related APE are limited.

This study aimed to examine the association between the simplified total Mastora score, as an indicator of thrombus burden and early mortality risk in patients with COVID-19-related APE.

## Methods

### Patients

A retrospective cohort study was conducted with patients aged 18 years and over, all diagnosed with COVID-19-related APE based on CTPA, between April 1, 2020, and April 1, 2021, at Ankara Bilkent City Hospital, a reference healthcare center with approximately 3,100 ward beds and 700 intensive care beds. Ethical approval for the study was obtained from the Ankara City Hospital Clinical Research Ethics Committee (approval number: E1-21-1835, date:June 9, 2021). The study was conducted in accordance with the principles of the Declaration of Helsinki.

### Data and definitions

Data were collected from electronic hospital records for all patients with COVID-19-related APE on gender, age, comorbidities, cancer history, medication use, and length of hospital stay. Laboratory tests were completed within 24 h of their diagnoses and included complete blood count, neutrophil-lymphocyte ratio (NLR), troponin I, brain natriuretic peptide (BNP), d-dimer, prothrombin time (PT), activated partial thromboplastin time (APTT), lactate dehydrogenase (LDH), C-reactive protein (CRP), procalcitonin, ferritin, interleukin-6, arterial blood gas, echocardiography, and lower extremity Doppler and CTPA findings. The dose and duration of the anticoagulant or thrombolytic therapy were recorded when available. In-hospital mortality was detected for all participants.

Patients with a COVID-19 diagnosis within 45 days before or 14 days after CTPA were considered to have COVID-19-related APE [14, 15]. COVID-19 diagnosis was based on either a positive SARS-CoV-2 reverse transcription-polymerase chain reaction (RT-PCR) test (i.e., PCR-confirmed) or thoracic CT findings of COVID-19, as defined by the British Society of Chest Radiology (i.e., radiological diagnosis) [16]. Patients with APE diagnoses after confirmation of COVID-19 were evaluated in the subgroup of post-COVID-19 APE.

The Wells score and the pulmonary embolism severity index (PESI) were calculated and classified, based on European Society of Cardiology (ESC) 2019 guidelines. Accordingly, early mortality risks (in-hospital or 30-day mortality) were classified as low, intermediate-low, intermediate-high, and high as proposed by ESC 2019 guideline [4]. “Early mortality risk” meets the definition of ESC 2019 guideline.

The World Health Organization considered severe COVID-19 disease when peripheral oxygen saturation (sPO_2_) value of < 90% in room air or a respiratory rate of > 30/min [17].

**Fig. 1:**
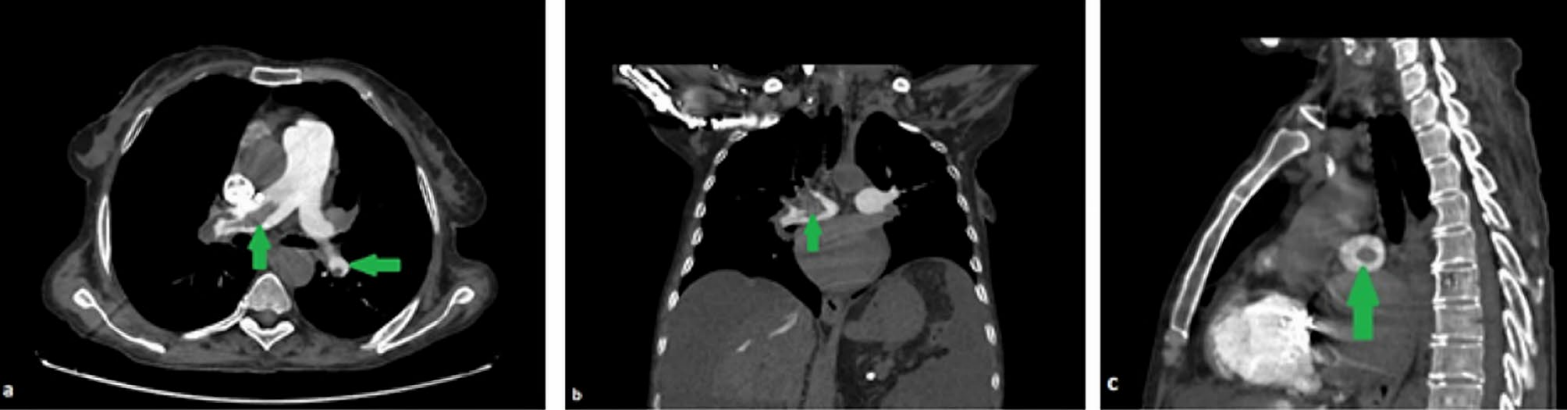
Computed tomography pulmonary angiography images of a 55-year-old female patient. Images showing a filling defect consistent with acute pulmonary thromboembolism causing < 50% stenosis in the right pulmonary artery in axial **(a)**, coronal **(b)**, and sagittal **(c)** sections (arrows)

**Fig. 2:**
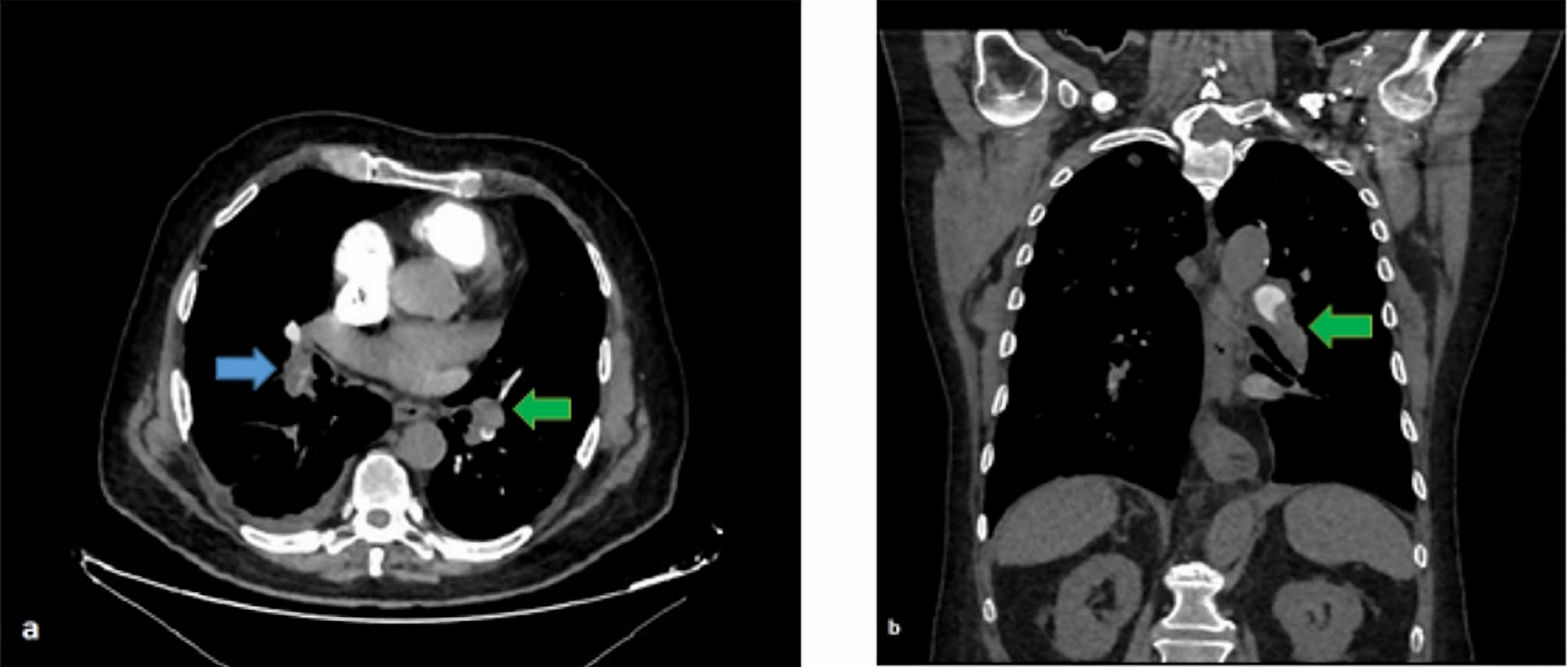
Computed tomography pulmonary angiography images of a 72-year-old male patient. A thromboembolic filling defect is observed to cause 50–99% stenosis in the lower lobar branches of both pulmonary arteries in the axial image(**a**, blue and green arrows).Coronal section view of the thrombus in the proximal left lower lobar branch(**b**, arrow)

**Fig. 3:**
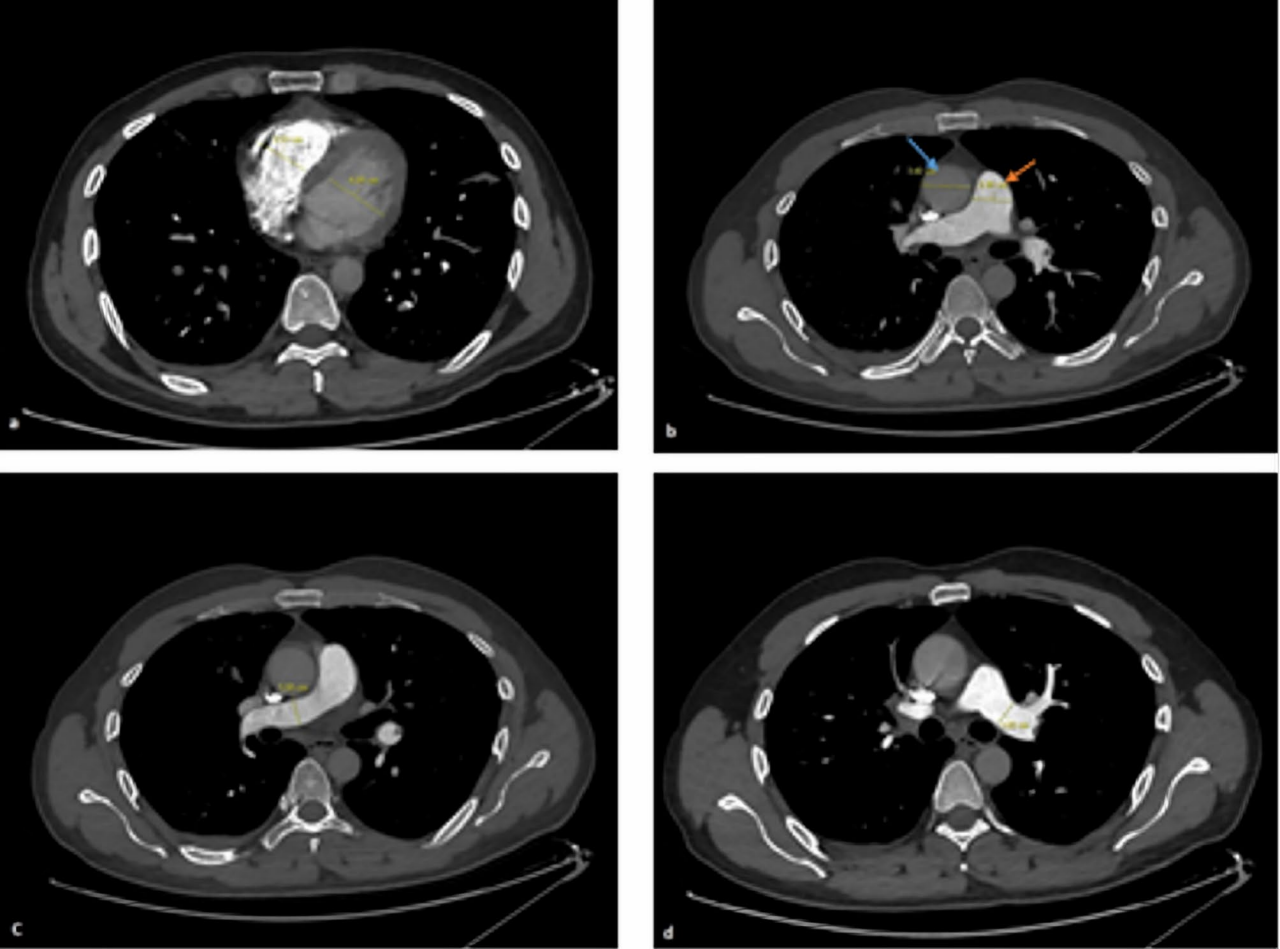
Measurement of the diameters in axial images. Measurement of the right and the left ventricle **(a)**, the ascending aorta and the pulmonary trunk **(b)**, the right **(c)**, and the left pulmonary artery **(d)** diameters in axial images

All CTPA images accessed from the Picture Archiving and Communication System (PACS) database of the hospital were re-examined by a thoracic radiology expert for confirmation of APE diagnosis. The localization of pulmoner embolism was grouped as pulmonary, lobar, segmental, subsegmental and extensive which was the embolism with involvement of multiple lobes with 25% or more of the entire pulmonary vascular bed [18]. The percentage of pulmonary artery occlusion due to pulmonary embolism was evaluated with the simplified Mastora score. Accordingly, five mediastinal, six lobar, and 20 segmental arteries were scored based on the percentage of occlusion. The entire pulmonary arterial bed was evaluated, with a score of 1 being assigned for < 50% stenosis, 2 for 50–99% stenosis, and 3 for total occlusion, with the highest score being 93 (Figs. 1 and 2). Diameters of the pulmonary trunk, both pulmonary arteries, superior vena cava, and right and left ventricles were further measured from the axial sections in each patient [6, 8](Fig. 3). The ratio of the main pulmonary artery diameter to the ascending artery diameter and that of the right ventricle (RV) diameter to the left ventricle (LV) diameter were calculated in millimeters.

### Statistical analysis

Categorical variables were presented as numbers and percentages. Categorical variables were compared with the Chi-square test. Quantitative variables are given as mean±standard deviation and median [interquartile range (IQR)]. Coefficient of variation (< 20%), kurtosis/standard error (< 1.96), skewness/standard error (< 1.96) ratios, visual (histogram and detrended Q-Q plot graphics) and analytical (Kolmogorov-Smirnov/Shapiro-Wilk tests) methods to evaluate the status of normal distribution. Kruskal-Wallis test was used for comparison of more than two independent groups. Pairwise comparisons were made with the Mann-Whitney U test, and the results were interpreted with the Bonferroni correction. The relationship between the simplified Mastora score and other quantitative variables was explored using the Spearman correlation coefficient (rho; ρ).

Multivariate logistic regression models were built to determine the factors associated with intermediate-high/high early mortality risk, with the total simplified Mastora score as the main effect and age, gender, comorbidity, presence of deep vein thrombosis, diagnosis with SARS-CoV-2 PCR, favipiravir, and hydroxychloroquine treatments as covariates. Model goodness of fit was tested with Hosmer-Lemeshow test. Model goodness of fit was not provided so methylprednisolone treatment, localization of embolism, and COVID-19 severity were excluded.

The performance of the simplified total Mastora score in predicting intermediate-high/high risk of early mortality was evaluated using the receiver operating characteristic (ROC) analysis. The value with the highest Youden index was determined as the optimal cut-off value for validity analysis.

The statistical significance level was taken as a p < 0.05(two-sided). No imputation was provided for the missing data. Statistical analyses were performed using the Statistical Package for the Social Sciences version 23(IBM SPSS^®^, Armonk, New York, USA) software package.

### Power analysis

Since there was no study with a similar design examining the relationship between the simplified total Mastora score and the risk of early mortality in APE, which was the objective of the current study, we performed the post-hoc power analysis with R version 3.6.1 software using the ‘*kwpower*’ command in the ‘*MultNonParam*’ package. We determined the power of the study as 99.96%. For binary logistic regression analysis, the power of the study was found to 88.37% via G*power 3.1 (Heinrich Heine Universität Düsseldorf, Düsseldorf, North Rhine-Westphalia, Germany).

## Results

Out of 1,988 patients consecutively diagnosed with APE between April 1, 2020, and April 1, 2021, at Ankara Bilkent City Hospital, 127 were hospitalized due to COVID-19-related APE. Of these 40 were excluded from analysis because Mastora scores could not be calculated, as CTPAs had been completed in outside facilities.

Of the total of 87 adult patients diagnosed with COVID-19-related APE 58 were males (66.7%) and the mean age was 62.5±16.2 years. The mean length of hospital stay was 12.9±10.8 days (median = 10.0, IQR = 9.0 days). Comorbidities were present in 59 (67.8%), of whom 12 had cancer. Of the 87 patients, 28 (32.2%) had hypertension, 20 (23.0%) had diabetes mellitus, 12 (13.8%) had malignancy, seven (8.0%) had coronary artery disease, five (5.7%) each had asthma and heart failure, three (3.4%) each had previous cerebrovascular events, Alzheimer’s disease, epilepsy, atrial fibrillation, obstructive sleep apnea syndrome, and chronic obstructive pulmonary disease, and one (1.1%) each had heart transplant recipient, bipolar disorder, psychosis, chronic kidney disease, goiter, gastritis, Down syndrome, cirrhosis, polycythemia vera, chronic venous insufficiency, autoimmune hepatitis, and previous tuberculosis. Twenty (23%) patients had deep vein thrombosis(DVT) in the lower extremities. Thirty-seven(42.5%) patients were diagnosed with concurrent APE and COVID-19, while 50(57.5%) were diagnosed with APE in the post-COVID-19 period. In the post-COVID-19 APE group, the median time from the diagnosis of COVID-19 to that of APE was 10.0(IQR = 13.0) days. Of all the patients included in the study, 10(11.5%) died. Table 1 presents the demographic, laboratory, and clinical characteristics of the study participants.

**Table 1:** Demographic, laboratory, and clinical characteristics of the studied patients

	n Mean ± Std. dev.	% Median (IQR)
Gender		
Male	58	66.7
Female	29	33.3
Age, years (n = 87)	62.5 ± 16.2	61 (24)
Presence of any comorbidity	59	67.8
Malignancy	12	13.8
COVID-19 diagnosis method		
SARS-CoV-2 PCR	59	67.8
Radiological examination (thoracic CT)	28	32.2
Presence of pneumonia	69	79.3
Presence of deep vein thrombosis	20	23
Echocardiogram findings		
Tricuspid valve regurgitation grade (n = 28)	1.46 ± 0.77	1.00 (1.00)
Pulmonary artery pressure (mmHg) (n = 29)	40.5 ± 12.9	38.0 (20.0)
PESI score (n = 87)	93.4 ± 15.1	17.0 (21.0)
PESI class		
I	21	24.1
II	21	24.1
III	13	14.9
IV	15	17.2
V	17	19.5
Total simplified Mastora score (n = 87)	19.5 ± 15.18	17.0 (21.0)
Early mortality risk groups		
Low	53	60.9
Intermediate-low	18	20.7
Intermediate-high/high	16	18.4
Laboratory findings		
Lymphocyte leucocytes (/µL) (n = 79)	1194.0 ± 748.6	1100 (1010)
Neutrophil/lymphocyte ratio (n = 76)	7.59 ± 7.65	5.65 (6.27)
Interleukin-6 (pg/ml) (n = 24)	28.5 ± 33.3	15.1 (24.8)
Troponin (ng/L) (n = 76)	48.0 ± 135.1	9.0 (29.0)
D-dimer (mg/L) (n = 72)	9.5 ± 10.3	5.3 (12.0)
Brain natriuretic peptide (ng/L) (n = 20)	2614 ± 5035	331 (1412)
Concurrent pulmonary embolism	37	42.5
Post-COVID-19 pulmonary embolism	50	57.5
Deceased	10	11.5

Std. dev: Standard deviation, CT: Computed tomography, IQR: Interquartile range, LV: Left ventricle, PAP: Pulmonary artery pressure, PESI: Pulmonary embolism severity index, PCR: Polymerase chain reaction, RV: Right ventricle

The distributions of the clinical characteristics of the patients according to the total Mastora score are shown in Table 2. The total Mastora score had statistically significant positive moderate correlations with pulmonary artery pressure (PAP) (ρ = 0.54, *p-value = 0.002*) and tricuspid valve regurgitation(ρ = 0.40, *p-value = 0.034*) and statistically significant positive weak correlations with troponin I(ρ = 0.32, *p-value = 0.005*), d-dimer (ρ = 0.30, *p-value = 0.011*), BNP(ρ = 0.26, *p-value = 0.005*), RV/LV ratio (ρ = 0.26, *p-value = 0.015*), and main pulmonary artery diameter (ρ = 0.22, *p-value = 0.041*). The total Mastora scores of all the patients included in the study and those diagnosed with post-COVID-19 APE, had statistically significant association with presence of PAP, presence of DVT, and early mortality risk (Table 2).

**Table 2:** Correlation of the total simplified Mastora score with clinical features, laboratory, and imaging findings

	Total Simplified Mastora Score			
	Overall (n = 87)		Post-COVID-19 APE (n = 50)	
	Rho (95% CI)	p-value	Rho (95% CI)	p-value
Age	0.03 (-0.19–0.25)	0.78	-0.06 (-0.34–0.23)	0.70
Wells score	0.07 (-0.15–0.28)	0.51	-0.01 (-0.29–0.28)	0.97
PESI score	0.03 (-0.19–0.24)	0.82	0.04 (-0.25–0.32)	0.79
PAP (mmHg)	0.54 (0.21–0.76)	**0.002**	0.65 (0.24–0.87)	**0.004**
Tricuspid valve regurgitation grade	0.40 (0.02–0.68)	**0.034**	0.44 (-0.07–0.77)	0.080
D-dimer (mg/L)	0.30 (0.07–0.50)	**0.011**	0.19 (-0.15–0.49)	0.26
Troponin (ng/L)	0.32 (0.09–0.51)	**0.005**	0.27 (-0.05–0.55)	0.087
BNP (ng/L)	0.26 (-0.22–0.64)	**0.005**	0.37 (-0.65–0.92)	0.47
NLR	-0.09 (-0.32–0.14)	0.43	-0.06 (-0.37–0.26)	0.69
Lymphocytes (/µL)	-0.01 (-0.23–0.22)	0.97	0.02 (-0.29–0.33)	0.90
CRP (mg/L)	-0.02 (-0.25–0.22)	0.88	0.14 (-0.19–0.44)	0.38
Procalcitonin (µg/L)	0.09 (-0.16–0.32)	0.47	0.16 (-0.17–0.47)	0.33
Interleukin-6 (pg/ml)	0.07 (-0.36–0.47)	0.75	0.10 (-0.49–0.63)	0.74
Systolic blood pressure	-0.03 (-0.27–0.21)	0.80	0.02 (-0.30–0.34)	0.89
Pulse oximetry-derived oxygen saturation	0.19 (-0.07–0.42)	0.14	0.11 (-0.23–0.43)	0.52
MPA diameter (mm)	0.22 (0.01–0.42)	**0.041**	0.23 (-0.07–0.48)	0.12
RPA diameter (mm)	0.10 (-0.12–0.31)	0.37	0.12 (-0.17–0.39)	0.42
LPA diameter (mm)	0.20 (-0.01–0.40)	0.058	0.21 (-0.07–0.47)	0.14
Ascending aorta diameter (mm)	0.09 (-0.13–0.30)	0.44	0.02 (-0.27–0.30)	0.92
SVC diameter (mm)	0.11 (-0.11–0.32)	0.30	-0.01 (-0.29–0.28)	0.96
MPA/ascending aorta ratio	0.12 (-0.10–0.33)	0.25	0.16 (-0.13–0.43)	0.25
RV/LV ratio	0.26 (0.05–0.46)	**0.015**	0.24 (-0.06–0.50)	0.10
	**Median total Mastora score (IQR)**	**p-value**	**Median total Mastora score (IQR)**	**p-value**
Gender		0.51		0.63
Male	16.5 (15.0)		14.0 (21.0)	
Female	18.0 (34.0)		11.0 (34.0)	
Presence of comorbidity	17.0 (22.0)	0.62	11.0 (23.0)	0.50
No comorbidity	15.5 (18.0)		15.5 (19.0)	
Diagnosed with SARS-CoV-2 PCR	13.0 (23.0)	0.054	13.5 (23.0)	0.30
Diagnosed with thoracic CT	18.0 (30.0)		20.0 (24.0)	
Pneumonia	17.0 (19.0)	0.50	15.0 (22.0)	0.40
Mild disease	12.0 (34.0)		6.0 (36.0)	
Patients with DVT	20.5 (28.0)	**0.013**	27.0 (36.0)	**0.023**
Patients without DVT	13.0 (22.0)		12.0 (20.0)	
Severe COVID-19	9.0 (25.0)	0.32	9.0 (43.0)	0.59
Non-severe COVID-19	17.0 (21.0)		18.0 (20.0)	
Early mortality risk		**0.002**		**0.009**
Low	11.0 (17.0)*		11.0 (18.0)*	
Intermediate-low	18.5 (18.0)		23.0 (28.0)	
Intermediate-high/high	31.5 (25.0)*		39.0 (19.0)*	

BNP: Brain natriuretic peptide, CRP: C-reactive protein, CT: Computed tomography, DVT: Deep venous thrombosis, IQR: Interquartile range, LPA: Left pulmonary artery, LV: Left ventricle, MPA: Main pulmonary artery, NLR: Neutrophil/lymphocyte ratio, PAP: Pulmonary artery pressure, PCR: Polymerase chain reaction, PESI: Pulmonary embolism severity index, APE: Acute pulmonary thromboembolism, RPA: Right pulmonary artery, RV: Right ventricle, SVC: Superior vena cava

The median total Mastora scores (IQR) were 11.0 (17.0), 18.0(18.5) and 31.5 (25.0) in the low, the intermediate-low risk and the intermediate-high/high risk groups, respectively *(p-value = 0.002*). In the post-hoc test, this difference was found to originate from the comparison of the low and intermediate-high/high risk groups (adjusted-*p-value = 0.002*). The analysis of the patients diagnosed with post-COVID-19 APE as a subgroup revealed a statistically significant difference between at least two groups (*p-value = 0.009*), and this was also explained by the difference between the low risk and intermediate-high/high risk groups (adjusted-*p-value = 0.017*).

Table 3 shows the distributions of the occluded vessel level, localization of embolism, medical treatments applied for COVID-19, and clinical characteristics according to the early mortality risk. The rate or mortality and favipiravir use statistically significantly differed between the three risk groups *(p-value = 0.012* and *p-value = 0.021*, respectively). Mortality rate was lower in the low risk group than the other 2 groups.

**Table 3:** Comparison of the early mortality risk groups according to the localization of embolism, COVID-19 treatment

	Early mortality risk					
	Low n (%)	Intermediate-low n (%)	Intermediate- high/high n (%)	p-value^†^	p-value^‡^	
Male gender	38 (71.7)	9 (50.0)	11 (68.8)	0.24	0.85	
Presence of pneumonia	42 (79.2)	15 (83.3)	12 (75.0)	0.87*	0.73*	
Presence of comorbidity	33 (62.3)	13 (72.2)	13 (81.3)	0.33	0.20	
Diagnosed with SARS-CoV-2 PCR	38 (71.7)	13 (72.2)	8 (50.0)	0.24	0.091	
Diagnosed with thoracic CT	15 (28.3)	5 (27.8)	8 (50.0)			
Presence of DVT	10 (18.9)	4 (22.2)	6 (37.5)	0.28*	0.19*	
Mortality	2 (3.8)	4 (22.2)	4 (25.0)	0.012*	0.081*	
Localization of embolism				0.051*	0.015*	
Pulmonary artery	14 (26.4)	8 (44.4)	5 (31.3)			
Lobar artery	18 (34.0)	5 (27.8)	4 (25.0)			
Segmental artery	16 (30.2)	3 (16.7)	2 (12.5)			
Subsegmental artery	4 (7.5)	1 (5.6)	-			
Extensive	1 (1.9)	1 (5.6)	5 (31.3)			
Unilateral	20 (37.7)	5 (27.8)	7 (43.8)	0.61	0.52	
Bilateral	33 (62.3)	13 (72.2)	9 (56.3)			
Favipiravir treatment	47 (88.7)	17 (94.4)	10 (62.5)	0.021*	0.012*	
HCQ treatment	12 (22.6)	5 (27.8)	7 (43.8)	0.27*	0.13*	
Methylprednisolone (> 250 mg/d)	4 (7.5)	4 (22.2)	1 (6.3)	0.22*	1.00*	
Median PESI score (IQR)	77.0 (36.0)	123.5 (56.0)	108.5 (21.0)	**< 0.001**	**0.021**	
Median MPA diameter, mm (IQR)	26.6 (5.0)	29.9 (4.8)	30.9 (6.1)	**0.013**	0.080	
Median RV/LV ratio (IQR)	1.03 (0.23)	1.13 (0.20)	1.35 (0.57)	**0.005**	**0.004**	
Median PAP, mmHg (IQR)	31.5 (11.0)	35.0 (-)	50.0 (16.0)	**0.003**	**< 0.001**	
Median tricuspid valve regurgitation grade (IQR)	1.0 (0.0)	1.0 (-)	1.75 (1.5)	**0.033**	**0.029**	
Median d-dimer, mg/L (IQR)	4.0 (6.0)	10.1 (25.0)	11.3 (19.0)	0.051	0.16	
Median troponin, ng/L (IQR)	6.0 (22.0)	13.0 (34.3)	20.5 (88.0)	**0.037**	**0.030**	
Median BNP, ng/L (IQR)	147.5 (617.0)	202.0 (1604.0)	877.0 (11280.0)	0.13	0.081	

BNP: Brain natriuretic peptide, CT: Computed tomography, DVT: Deep venous thrombosis, HCQ: Hydroxychloroquine, IQR: Interquartile range, LV: Left ventricle, MPA: Main pulmonary artery, PAP: Pulmonary artery pressure, PCR: Polymerase chain reaction, PESI: Pulmonary embolism severity index, RV: Right ventricle,

* Fisher’s exact test,

^**†**^ Low vs. Intermediate-low vs. Intermediate-high/high early mortality risk,

^**‡**^ Low / Intermediate-low vs. Intermediate-high/high early mortality risk

With the multiple logistic regression model, intermediate-high/high early mortality risk was associated statistically significantly with total simplified score (adj OR = 1.06, 95%CI = 1.02–1.11,*p-value = 0.008*), and favipiravir treatment (adj OR = 0.13, 95%CI = 0.02–0.93, *p-value = 0.041*)(Table 4).

**Table 4:** Factors associated with the intermediate-high/high early mortality risk groups, binary logistic regression

	Univariate analysis			Multivariate analysis		
	cOR	95% CI	p-value	aOR	95% CI	p-value
Total simplified Mastora score	1.06	1.02–1.10	**0.003**	1.06	1.02–1.11	**0.008**
Male gender	1.12	0.35–3.61	0.85	2.04	0.42–10.00	0.38
Age (years)	1.02	0.98–1.05	0.31	1.02	0.97–1.06	0.51
Presence of comorbidity	2.36	0.61–9.05	0.21	3.91	0.65–23.26	0.14
Patients with DVT	2.44	0.76–7.86	0.13	2.36	0.55–10.25	0.25
Diagnosed with SARS-CoV-2 PCR	0.39	0.13–1.19	0.098	0.93	0.23–3.83	0.92
Favipiravir treatment	0.18	0.05–0.65	**0.009**	0.13	0.02–0.93	**0.041**
HCQ treatment	2.47	0.80–7.63	0.12	0.87	0.16–4.62	0.87

cOR: crude odds ratio, aOR: adjusted odds ratio, CI: Confidence interval,

DVT: Deep vein thrombosis, SARS-CoV-2: Severe acute respiratory syndrome-Coronavirus-2

PCR: Polymerase chain reaction, HCQ: Hydroxychloroquine

The analysis was completed with 87 patients. Hosmer-Lemeshow p-value = 0.19, Nagelkerke R^2^ = 0.348

**Fig. 4:**
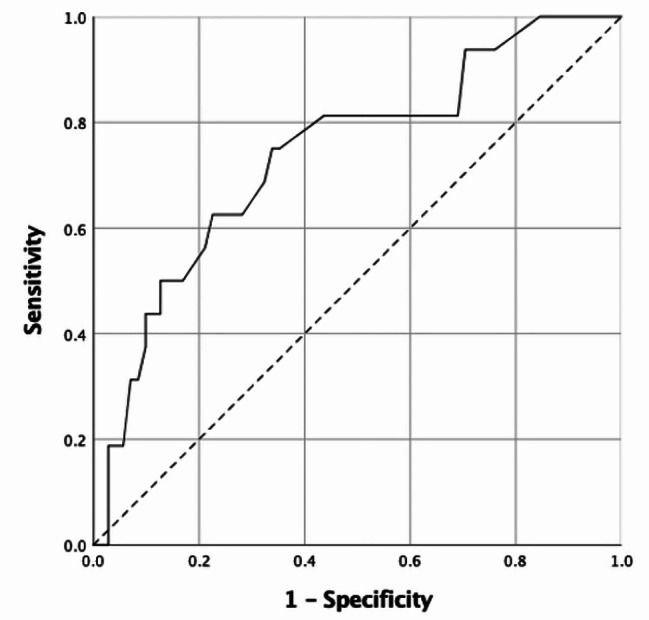
Receiver operating characteristic curve of the total simplified Mastora score in the prediction of intermediate-high/high risk of early mortality

The validity of the simplified total Mastora score for prediction of the intermediate-high/high risk of early mortality was high [AUC = 0.740(95%CI = 0.603–0.877)] (Fig. 4). When the optimal cut-off value was taken as 18.5, this score had a sensitivity of 75.0%, specificity of 66.2%, positive predictive value of 33.3%, negative predictive value of 92.2%, and total accuracy of 67.8%.

## Discussion

To the best of our knowledge, this is the first study to examine the relationship of thrombus burden with clinical features, disease severity, and early mortality risk in patients diagnosed with COVID-19-related APE. The results indicated a significant association between the total Mastora score calculated using CTPA and early mortality risk in patients diagnosed with COVID-19-related APE. The patients in the intermediate-high/high risk group had a significantly higher total simplified Mastora score than those in the low risk group.

In a study from China using the 2014 ESC criteria, the mean total Mastora score was 31.9±8.9 in the high risk group and 10.2±8.2 in the remaining patients in a cohort of 120 patients (*p-value < 0.001*) [19]. In a retrospective cohort study including 100 patients with APE in Türkiye, risk stratification according to the American Heart Association (AHA) 2014 guideline showed a positively correlation with the simplified Mastora score (*p-value < 0.001*) [20]. These data mostly indicate a positive correlation between mortality or risk of mortality and the Mastora score. Since the massive-submassive and low-risk groups proposed in the AHA 2014 guideline can be used similarly to the ESC 2019 guideline’s high, intermediate, and low early mortality risk groups, the results of this study were considered comparable [21].

Our results showed a positive correlation between the total simplified Mastora score and the main pulmonary artery diameter, RV/LV ratio, pulmonary artery pressure, degree of tricuspid valve regurgitation, and d-dimer, troponin I, and BNP levels, which are important parameters clinically used to assess the severity of APE. However, the Mastora score did not have any correlation with PESI, a score that indicates the severity of pulmonary embolism. When the patients in the post-COVID-19 APE group (n = 50) were evaluated as a subgroup, the simplified total Mastora score was statistically significantly different according to the early mortality risk. PAP and the presence of DVT were determined as other associated factors. There was also a similar correlation between the simplified Mastora score and other factors associated with APE in the overall cohort group, although not in the post-COVID-19 APE group. Furthermore, when the whole sample was considered, the simplified Mastora score had a statistical significance level similar to those of the remaining factors, except the BNP level. Since the post-COVID-19 APE group was formed to perform a subgroup analysis, and it had a smaller sample size, we consider that the results of the post-COVID-19 APE group and the overall cohort can be evaluated together in terms of the simplified total Mastora score.

The simplified total Mastora score had a statistically significant positive correlation with the ratio of the RV/LV diameters used in the evaluation of right ventricular dysfunction in the presence of APE. A similar positive correlation (Kendall-tau = 0.24) was reported in a study from Belgium evaluating 80 patients presenting to the emergency department with APE [22]. In other publications, the coefficient of correlation was reported to be 0.36 in 65 patients in Germany [23] and 0.675 in a multicenter study from China, in which 115 patients were evaluated [24]. This finding is generally consistent in the literature and indicates an increase in right heart dysfunction with the increase of thrombus burden.

We found no correlation between the simplified total Mastora score and PESI used to determine the severity of pulmonary embolism and the Wells score developed to determine the probability of a pulmonary embolism diagnosis. Similarly, in a study in which 246 patients were evaluated in Leipzig, Germany, no correlation was detected between the Geneva scores used for similar purposes and the PESI and Wells scores [10]. A similar finding was reported in a study conducted with 100 patients in Türkiye, with the simplified Mastora score having no significant correlation with the Wells score or the modified PESI [20]. It is considered that the absence of these correlations contrary to expectations but consistent with the previous studies is due to the presence of many variables affecting the severity of the disease, in addition to thrombus burden. Furthermore, determining the risk of early mortality risk from different aspects will make the estimation more powerful.

D-dimer is one of the fibrin degradation products assisting clinicians in diagnosis, although it is not specific. Since the simplified total Mastora score is a scoring system related to thrombus burden, it was found to have a positive correlation with d-dimer, as expected. In a retrospective cohort study conducted in Türkiye, it was reported that the simplified Mastora score and the d-dimer level were correlated (r = 0.300, *p-value = 0.002*) [20]. In a study by Lerche et al. evaluating 246 patients in Germany, such a relationship was not found, but the result was close to statistical significance (ρ = 0.15, *p-value = 0.09*) [10]. This absence of statistical significance and the lower coefficient may be potentially related to the d-dimer level being evaluated at a later stage of the disease. On the other hand, in a study conducted with 69 patients in a university hospital in China, the d-dimer level was positively correlated with the Mastora score (r = 0.417, *p-value < 0.001*) [25]. Interestingly, in both studies, blood samples were collected within 24 h of presentation to the hospital or diagnosis in the post-COVID-19 group (after CTPA). It is suggested that the d-dimer level peaks within one week, and then decreases, and therefore the time of d-dimer testing may be important in determining this relationship [26]. In a single-center prospective study in New York, there was a moderate positive correlation between the total Mastora score and the d-dimer level in 23 COVID-19-related pulmonary embolism cases (ρ = 0.61, *p-value = 0.002*) [27]. We observed a similar relationship in the COVID-19-related APE subgroup.

In a study investigating whether the total simplified Mastora score predicted APE with a high risk of early mortality, the AUC value was found to be 0.968 (95%CI = 0.942–0.994). Using the Youden index, the authors determined the optimal cut-off value as 19.35 [19]. Although this value is similar to the value found in our study, it is higher. We may have obtained a slightly lower cut-off value due to predict the intermediate-high/high risk group of early mortality together. The reason why we aimed to predict the intermediate-high risk group in addition to the high-risk group in this study is that the former also needs to be closely followed up in terms of progression, and although there is no routine thrombolytic therapy recommendation in the intermediate-high risk group as in the high-risk group, treatment can still be applied in selected cases [4].

Retrospective design limited the number of variables to those recorded in the electronic database, yet, potential biases due to memory and self-report were avoided. Misclassification bias was avoided by re-evaluations of all CTPA images by a radiology expert blinded to the clinical characteristics of the patients. Restricting study participants to hospitalized cases could have led to selection bias favoring severe cases, yet, almost all COVID-19 cases in Türkiye were hospitalized over the time period studied. Generalizability of our results is limited due to the single-center and hospital-based design, though. This study has the statistical power to show the association between early mortality risk groups and simplified total Mastora score. However, it is underpowered to detect an association with death, possibly due to the lack of 30-day out-of-hospital early mortality data.

## Conclusions

The simplified total Mastora score, an indicator of pulmonary vascular thrombus burden in patients diagnosed with COVID-19-related APE, was found to be higher in the intermediate-high/high risk groups. It is recommended to predict the early mortality risk of patients with APE by calculating the simplified total Mastora score using CTPA performed for diagnostic purposes and to use the information obtained from the widely accepted and available tools in the close follow-up and treatment decisions of these patients.

## Data Availability

The datasets used and/or analyzed during the current study are available from the corresponding author upon reasonable request.

## List of abbreviations

adj: adjusted
AHA: American Heart Association
APE: Acute pulmonary thromboembolism
aPTT: activated partial thromboplastin time
AUC: Area under the curve
BNP: Brain natriuretic peptide
COVID-19: Coronavirus 2019 Disease
CRP: C-reactive protein
CT: Computed tomography
CTPA: Computed tomography pulmonary angiography
DVT: Deep vein thrombosis
ESC: European Society of Cardiology
IQR: Interquartile range
LDH: Lactate dehydrogenase
LV: Left ventricle
OR: Odds ratio
PAP: Pulmonary artery pressure
PESI: Pulmonary embolism severity index
PT: Prothrombin time
ROC: Receiver operating characteristic
RT-PCR: Real time-polymerase chain reaction
RV: Right ventricle
